# On the problem of the catatonic disorders taxonomy

**DOI:** 10.1192/j.eurpsy.2022.1994

**Published:** 2022-09-01

**Authors:** V. Lobanova, A. Smulevich, E. Voronova

**Affiliations:** 1 Mental Health Research Center, Department Of Borderline Mental Pathology And Psychosomatic Disorders, Moscow, Russian Federation; 2 Mental Health Research Center, Department Of ‘borderline’ Psychiatry And Psychosomatic Disorders, Moscow, Russian Federation

**Keywords:** Catatonia, schizophrenia mental disorders, schizophrénia, movement disorders

## Abstract

**Introduction:**

In accordance with the systematics of modern international clinical guidelines (DSM-V, ICD-11), catatonia is qualified as a transnosological formation, which boundaries expandes by including non-psychotic movement disorders (hysterical, affective, negative, etc.). This study presents the psychopathological systematics of movement disorders, based on a new dimensional model of catatonia.

**Objectives:**

60 patients with an established diagnosis of schizophrenia or SSD (F20, F21, F25.01, F25.11, F25.21, F25.22), catatonic disorders in the structure of which persist throughout the course of the disease or determine the clinical picture of phases.

**Methods:**

Clinical, psychometric (BFCRS, SANS, SAPS, HADS), statistic.

**Results:**

Three catatonic syndromes (S.) have been identified. 1. S. of stereotypical catatonia - presented by the mechanism of affiliation with negative symptoms (R between BFCRS Total Score (TS) and Avolition-Apathy SANS – 0,875): tendency to stereotypical activity; general, increasing slowness (SANS avolition-apathy -2,9±0,5; BFCRS TS – 11,1±0,2). 2. S. of parakinetic catatonia - includes paroxysms formes by the mechanism of mental automatism (with the loss of motor acts voluntary effect ) (R BFCRS TS/Persecutory Delusions SAPS– 0,764): irregular polymorphic movement disorders of hyperkinetic and akinetic types, impulsive actions, akinesias (Persecutory Delusions - 2,3±0,4; BFCRS – 19,5±2,3). 3. Affective - catatonic S. - including both the lightest (at the level of recurrent depression) variants of affective-catatonic phases (R BFCRS TS/HADS – 0,732; BFCRS – 5,1±0,4; HADS -15,1±2,4), and more severe affective-catatonic states based on schizoaffective psychoses (R BFCRS TS/SAPS TS– 0,783; BFCRS – 15,3±2,1; SAPS – 3,1±0,2).

**Conclusions:**

Catatonia is not a single dimension, represented by heterogeneous movement disorders, differing both in the mechanism of formation and in the psychopathological structure.

**Disclosure:**

No significant relationships.

